# Outcomes in patients with relapsed/refractory multiple myeloma with extramedullary disease: a meta-analysis

**DOI:** 10.1007/s00277-025-06705-3

**Published:** 2025-12-03

**Authors:** Peter M. Voorhees, Shaji Kumar, Saad Z. Usmani, Jing Christine Ye, Yael C. Cohen, Emma Scott, Robin L. Carson, Christoph Heuck, Ryan Gan, Benjamin Ackerman, Jenny Zhang, Eleanor Caplan, Trilok Parekh, María-Victoria Mateos

**Affiliations:** 1 Department of Hematologic Oncology and Blood Disorders, Atrium Health Levine Cancer Institute, Wake Forest University School of Medicine, 1021 Morehead Medical Dr., Building 2, Charlotte, NC 28204 USA; 2 Department of Hematology, Mayo Clinic Rochester, Rochester, MN USA; 3 Department of Medicine, Memorial Sloan Kettering Cancer Center, New York, NY USA; 4 MD Anderson Cancer Center, University of Texas, Houston, TX USA; 5 Tel-Aviv Sourasky (Ichilov) Medical Center, Tel Aviv, Israel; 6 Faculty of Medical & Health Sciences, Tel Aviv University, Tel Aviv, Israel; 7 Johnson & Johnson, Spring House, PA USA; 8 Johnson & Johnson, Raritan, NJ USA; 9 Johnson & Johnson, Titusville, NJ USA; 10 University Hospital of Salamanca, IBSAL/CIC/CIBERONC, Salamanca, Spain

**Keywords:** Bayesian modeling, Extramedullary disease, Meta-analysis, Meta-regression analysis, Relapsed/refractory multiple myeloma

## Abstract

Extramedullary disease (EMD), an aggressive form of multiple myeloma (MM), may require a multi-targeted treatment approach rather than standard MM therapies. While precise EMD treatment outcome estimates remain challenging given small clinical trial patient numbers, pooled estimates across studies may increase outcome precision. Meta-regression analyses used Bayesian multilevel, random-effects modeling of patients with and without EMD across nine historical clinical studies of standard treatment regimens, including daratumumab, for relapsed/refractory multiple myeloma (RRMM) from 2013 to 2019. EMD was defined as soft tissue plasmacytomas noncontiguous with bone (“true” EMD). Adjustments were performed to account for differences in baseline age, number of prior lines of therapy (LOT), and International Staging System (ISS) stage. Outcomes included overall response rate (ORR), progression-free survival (PFS), and overall survival (OS). In patients with EMD (*n* = 158) versus without EMD (*n* = 2706), pooled ORR (95% credible interval) was 20.7% (11.7–33.9) versus 66.2% (53.0–77.4) with an odds ratio of 0.13 (0.09–0.20), pooled median PFS was 6.3 (4.2–9.5) versus 12.9 (8.8–18.8) months with a hazard ratio of 1.95 (1.63–2.32), and pooled median OS was 21.0 (15.9–27.9) versus 39.0 (31.0–48.5) months with a hazard ratio of 1.87 (1.53–2.26). Poorer outcomes in patients with versus without EMD were consistent following adjustment for age, number of prior LOT, and ISS stage. These results in patients with RRMM treated with standard treatment regimens confirmed notably worse outcomes in patients with versus without EMD, emphasizing continued unmet clinical need in this patient population.

**Supplementary Information:**

The online version contains supplementary material available at 10.1007/s00277-025-06705-3.

## Introduction

Multiple myeloma (MM) is a hematologic malignancy characterized by aberrant plasma cell proliferation within the bone marrow microenvironment [1, 2]. While the bone marrow microenvironment is intrinsic to the initial proliferation of plasma cells, clones or subclones of malignant plasma cells, termed plasmacytomas, can grow independent of the bone marrow in soft tissues and other organs; i.e., plasmacytomas that neither arise from the bone nor disrupt the cortex of the bone are termed extramedullary disease (EMD; “true” EMD), while plasmacytomas extending from within the bone are termed paramedullary plasmacytomas [3–5].

The median time from MM diagnosis to EMD occurrence is approximately 19 to 23 months, by which time many patients have relapsed on standard treatments; in patients with relapsed/refractory MM (RRMM), EMD incidence rates are higher (3.4%–14.0%) than those observed in patients with newly diagnosed MM (0.5%–4.8%) [1, 6–8]. These higher incidence rates are likely due to the improved survival of patients with MM, resulting from more effective treatments (proteasome inhibitors [PIs], immunomodulatory drugs [IMiDs], anti-CD38 monoclonal antibodies [mAbs], and novel immunotherapies including chimeric antigen receptor [CAR]-T cell therapies and bispecific antibodies) [9–12], as well as due to improved sensitivity of imaging approaches with increased use of positron emission tomography-computed tomography and magnetic resonance imaging scans [1, 6, 13–15]. While outcomes in patients with RRMM remain poor, EMD is associated with particularly dismal patient outcomes due to its association with high-risk cytogenetics, evasion of cellular apoptosis, and therapeutic resistance [3, 4]. Patients with EMD have poorer outcomes versus patients with paramedullary plasmacytomas [6, 14, 16, 17].

There is currently no consensus on the treatment of patients with EMD [15]. Generally, patients with EMD who are treated with standard treatment regimens (including chemotherapy, PIs, IMiDs, and anti-CD38 mAbs) have low overall response rates (ORRs) and a short median progression-free survival (PFS), with ORRs ranging from 5 to 35% and median PFS of approximately 1.4 to 4.5 months [18–23]. A pooled analysis from the prospective LocoMMotion (NCT04035226) and MoMMent (NCT05160584) studies showed that patients with triple-class exposed (to a PI, IMiD, and anti-CD38 mAb) RRMM with EMD, who were enrolled between 2019 and 2022 and received standard regimens, had substantially poorer survival outcomes than patients without EMD, with median PFS and median overall survival (OS) both roughly halved in patients with versus without EMD [24]. As such, there remains a substantial unmet need for effective treatments that offer clear benefits to patients with EMD.

Across clinical studies, response outcomes are generally reported only in small numbers of patients with EMD, and EMD definitions can include mixes of both EMD and paramedullary plasmacytomas; as such, response outcomes vary across studies, resulting in imprecise estimates of outcomes for standard therapies among patients with RRMM with EMD. Therefore, generating aggregated estimates across studies using a meta-analytic framework may better account for cross-study variability and may improve the precision of estimates of outcomes in this patient population.

We report pooled outcomes of patients with versus without EMD by conducting meta-analyses of relevant clinical studies between 2002 and 2024.

## Methods

### Study selection and data sources

Clinical studies of standard-of-care and novel therapies approved by the US Food and Drug Administration (FDA), including daratumumab, and listed in the National Comprehensive Cancer Network guidelines [25] were identified. Johnson & Johnson-conducted internal clinical studies (referred to as “historical” throughout) of these therapies in patients with RRMM and EMD (defined as soft tissue plasmacytomas noncontiguous with bone or “true” EMD) at baseline were selected for inclusion in the analyses. A recently published systematic literature review of randomized clinical studies that detailed therapies for RRMM from January 1, 2002 to February 28, 2022 [26] was then leveraged to identify relevant external studies (referred to as “published” throughout) for inclusion in the analyses. An additional review of available literature was conducted to identify clinical studies that would not have been captured in the published systematic literature review, either due to timing (i.e., were published between March 2022 and July 2024) or due to status as nonrandomized, single-arm clinical studies. Current analyses focused on common off-the-shelf treatment options for RRMM, including PIs, IMiDs, and anti-CD38 mAbs; studies assessing the efficacy of CAR-T cell and bispecific antibody therapies were not included.

Data from nine historical studies were included in a meta-regression analysis to compare outcomes in patients with versus without EMD. Data from these studies were also included in a meta-analysis inclusive of patients with EMD only. Historical studies included were the NCT02852837 phase 1 study; PAVO phase 1b study; SIRIUS phase 2 study; and POLLUX, CASTOR, LEPUS, APOLLO, COLUMBA, and CANDOR phase 3 studies (Table 1), with enrollment initiation ranging from 2013 to 2019. Data collection and follow-up were per individual study protocols.

**Table 1:** Clinical studies included in analyses

**Historical studies**			
**NCT number**	**Study name**	**Phase**	**Treatment arms**
NCT02852837	Not available	1	Daratumumab IV
NCT02519452	PAVO	1b	Daratumumab SC with rHuPH20
NCT01985126	SIRIUS	2	Daratumumab IV
NCT03180736	APOLLO	3	Pomalidomide, dexamethasone, daratumumab (DpD) and pomalidomide, dexamethasone (Pd)
NCT03158688	CANDOR	3	Carfilzomib, dexamethasone, daratumumab (DKd) and carfilzomib, dexamethasone (Kd)
NCT02136134	CASTOR	3	Daratumumab, bortezomib, dexamethasone (DVd) and bortezomib, dexamethasone (Vd)
NCT03277105	COLUMBA	3	Daratumumab IV or SC
NCT03234972	LEPUS	3	Daratumumab, bortezomib, dexamethasone (DVd) and bortezomib, dexamethasone (Vd)
NCT02076009	POLLUX	3	Daratumumab, lenalidomide, dexamethasone (DRd) and lenalidomide, dexamethasone (Rd)
**Published studies**			
**NCT number**	**Study name**	**Phase**	**Treatment arms**
NCT02336815	STORM	2	Selinexor and dexamethasone
NCT02990338	ICARIA	3	Pomalidomide, dexamethasone, isatuximab (Isa-Pd) and pomalidomide, dexamethasone (Pd)
NCT03275285	IKEMA	3	Carfilzomib, dexamethasone, isatuximab (Isa-Kd) and carfilzomib, dexamethasone (Kd)

Abbreviations of treatment regimens are included in parenthesis where appropriate

*IV* intravenous, *NCT* National Clinical Trial, *rHuPH20* recombinant human hyaluronidase, *SC* subcutaneous

As the definition of plasmacytomas as extramedullary (“true” EMD that is soft tissue only and noncontiguous with bone) or paramedullary (contiguous with bone) differed between the historical and published studies reported herein, data from an additional three published clinical studies (comprising a mix of patients with both extramedullary and paramedullary disease) were included in a sensitivity meta-analysis only. Published studies included were the STORM phase 2 study and the ICARIA and IKEMA phase 3 studies (Table 1). Published studies were determined suitable for inclusion if outcomes were reported by EMD status at baseline (including both EMD and paramedullary plasmacytomas) for at least ORR, PFS, or OS. The denominator and either the number or percentage of patients with EMD who achieved a partial response or better were required for ORR. A Kaplan–Meier plot with a life table or indicators of censored patients was required for PFS, OS, and duration of response (DOR) across all published studies to approximate observed data. Individual patient-level data were reconstructed for PFS, OS, and DOR in patients with EMD following defined algorithms [27, 28].

This report included three separate analyses (i.e., the meta-regression analysis, meta-analysis, and sensitivity meta-analysis) to enable a thorough assessment of data reliability and robustness. Specifically, the meta-regression analysis explored data heterogeneity among patients with and without EMD, the meta-analysis improved overall sample population size by pooling estimates across studies, and the sensitivity meta-analysis included additional published studies and model assumptions; together, these analyses complemented one another and provided confidence in the model and results.

### Outcomes of interest

The primary outcome of interest was ORR defined as the proportion of patients who achieved a partial response or better per International Myeloma Working Group (IMWG) 2016 criteria [29]. ORR was reported as recorded in the historical clinical study data or within the published studies. Secondary outcomes of interest were PFS and OS. PFS was defined as time to progression per IMWG 2016 criteria [29] or death, whichever came first. OS was defined as time from first study treatment dose to death due to any cause. Patients were censored if they reached the end of the study period or were lost to follow-up. Outcomes were pooled across all studies included in each analysis.

### Analyses

Meta-analyses were performed using a Bayesian multilevel, random-effects model fit to patient-level data across nine historical clinical studies of patients with and without EMD [30]. To account for variability within and between the historical clinical studies, random effects were specified for the treatment by the study indicator variable. For the meta-regression analysis, EMD status was included as a fixed-effect variable and study treatment was included as a random-effect variable (reported as the “base model” results). Adjustments were made for the baseline prognostic variables of patient age, number of prior lines of therapy (LOT), and International Staging System (ISS) stage to account for differences in baseline characteristics. Age and number of prior LOT were modeled as continuous variables using splines to allow for potential nonlinear relationships with the outcomes, while ISS stage was modeled as a factor. Data from the three published clinical studies, as available for each of the outcomes of interest, were included in a sensitivity meta-analysis to include additional newer standard therapies (isatuximab and selinexor) and model assumptions.

All patients within the clinical studies who met the inclusion criteria were included in the analysis; no sample size or power calculations were conducted. Baseline characteristics for continuous data were summarized using descriptive statistics. Categorical data were summarized by counts and proportions. For ORR, a Bernoulli model with a logit-link was used along with priors on the population-level intercept and on the random intercepts [30]. For PFS, OS, and DOR, a Weibull model with a log-link was used to account for right-censored times, along with priors on the population-level intercept and on the random intercepts [31].

Posterior distributions were used to estimate median and 95% credible intervals (CIs) for proportion of responders, median time to event, and survival curves. Response rates and odds ratios were reported for ORR, and medians with hazard ratios were reported for PFS, OS, and DOR. Data transformations and modeling were conducted using R software (v4.4.2). Code and analysis results were reviewed independently and verified by two additional statisticians.

## Results

### Patient information and baseline demographics

Across nine historical clinical studies analyzed, 158 patients had EMD, and 2706 patients did not have EMD (Tables 2 and 3; Table S1). Among the 158 patients, 89 (56%) were male and median ages ranged from 53 to 74 years (Table 2). In eight studies, the majority of patients (≥ 50%) with EMD had an Eastern Cooperative Oncology Group performance status of 1, and in six studies, most patients (≥ 75%) self-identified as White. In comparison, patients without EMD generally were of a similar age, had a comparable number of prior LOT, and had comparable proportions of patients with ISS stage I or II disease (Tables 2 and 3).

**Table 2:** Baseline characteristics across studies in patients with RRMM with EMD

Characteristics	APOLLO *N* = 23	CANDOR *N* = 21	CASTOR *N* = 23	COLUMBA *N* = 35	LEPUS *N* = 19	MMY1003 *N* = 2	PAVO *N* = 4	POLLUX *N* = 15	SIRIUS *N* = 16
Median age, years (IQR)	59 (55–71)	62 (55–71)	63 (58–67)	63 (57–70)	62 (54–65)	53 (45–60)	74 (66–79)	63 (56–68)	59 (54–64)
Age ≥ 65 years, *n* (%)	8 (35)	8 (38)	9 (39)	16 (46)	6 (32)	0 (0)	3 (75)	7 (47)	2 (13)
Female, *n* (%)	7 (30)	9 (43)	12 (52)	18 (51)	6 (32)	1 (50)	1 (25)	7 (47)	8 (50)
Race, *n* (%)									
White	22 (96)	17 (81)	19 (83)	32 (91)	0 (0)	0 (0)	3 (75)	7 (47)	15 (94)
Other	0 (0)	4 (19)	4 (17)	2 (6)	19 (100)	2 (100)	0 (0)	7 (47)	1 (6)
Missing	1 (4)	0 (0)	0 (0)	1 (3)	0 (0)	0 (0)	1 (25)	1 (6)	0 (0)
Baseline ECOG PS, *n* (%)									
0	11 (48)	7 (33)	6 (26)	8 (23)	6 (32)	0 (0)	0 (0)	6 (40)	2 (13)
1	7 (30)	11 (53)	12 (52)	19 (54)	11 (58)	1 (50)	3 (75)	8 (53)	12 (75)
2	5 (22)	3 (14)	5 (22)	8 (23)	2 (11)	1 (50)	1 (25)	1 (7)	2 (13)
Missing	0 (0)	0 (0)	0 (0)	0 (0)	0 (0)	0 (0)	0 (0)	0 (0)	0 (0)
Prior ASCT, *n* (%)	16 (70)	11 (52)	12 (52)	24 (69)	2 (11)	0 (0)	1 (25)	10 (67)	16 (100)
Number of prior LOT, *n* (%)									
1	1 (4)	9 (43)	14 (61)	0 (0)	8 (42)	0 (0)	0 (0)	6 (40)	0 (0)
2	19 (83)	6 (29)	2 (8)	0 (0)	4 (21)	0 (0)	1 (25)	9 (60)	1 (6)
3	3 (13)	6 (29)	5 (22)	8 (23)	3 (16)	1 (50)	0 (0)	0 (0)	1 (6)
≥ 4	0 (0)	0 (0)	2 (9)	27 (77)	4 (21)	1 (50)	3 (75)	0 (0)	14 (88)
IgG subtype MM, *n* (%)	11 (48)	11 (52)	10 (43)	19 (54)	11 (58)	0 (0)	1 (25)	3 (20)	11 (69)
Cytogenetic profile,^a^* n* (%)									
High	3 (13)	1 (5)	6 (26)	1 (3)	9 (47)	0 (0)	0 (0)	0 (0)	2 (13)
Standard	11 (48)	9 (43)	11 (48)	23 (66)	10 (53)	2 (100)	2 (50)	10 (67)	12 (75)
Missing	9 (39)	11 (52)	6 (26)	11 (31)	0 (0)	0 (0)	2 (50)	5 (33)	2 (13)
ISS stage, *n* (%)									
I	11 (48)	5 (24)	8 (35)	11 (31)	8 (42)	1 (50)	0 (0)	9 (60)	3 (19)
II	6 (26)	10 (48)	9 (39)	16 (46)	6 (32)	0 (0)	3 (75)	3 (20)	6 (38)
III	6 (26)	6 (28)	6 (26)	8 (23)	5 (26)	1 (50)	1 (25)	3 (20)	7 (44)
Missing	0 (0)	0 (0)	0 (0)	0 (0)	0 (0)	0 (0)	0 (0)	0 (0)	0 (0)

^a^Cytogenetic profile definition may vary across studies

*ASCT* autologous stem cell transplant, *ECOG PS* Eastern Cooperative Oncology Group performance status, *EMD* extramedullary disease, *IgG* immunoglobulin-G, *IQR* interquartile range, *ISS* International Staging System, *LOT* line of therapy, *MM* multiple myeloma, *RRMM* relapsed/refractory multiple myeloma

**Table 3:** Baseline characteristics across studies in patients with RRMM without EMD

Characteristics	APOLLO *N* = 281	CANDOR *N* = 445	CASTOR *N* = 475	COLUMBA *N* = 487	LEPUS *N* = 192	MMY1003 *N* = 48	PAVO *N* = 116	POLLUX *N* = 554	SIRIUS *N* = 108
Median age, years (IQR)	59 (55–71)	64 (58–70)	64 (57–70)	67 (60–73)	61 (54–67)	61 (53–65)	66 (60–72)	65 (59–71)	65 (58–70)
Age ≥ 65 years, *n* (%)	173 (62)	218 (49)	232 (49)	285 (59)	72 (38)	12 (25)	70 (60)	289 (52)	56 (52)
Female, *n* (%)	136 (48)	189 (42)	201 (42)	219 (45)	78 (41)	25 (52)	65 (56)	225 (41)	52 (48)
Race, *n* (%)									
White	250 (89)	349 (78)	416 (88)	376 (77)	0 (0)	0 (0)	88 (76)	386 (70)	86 (80)
Other	4 (1)	96 (22)	48 (10)	86 (18)	192 (100)	48 (100)	12 (10)	112 (20)	19 (18)
Missing	27 (10)	0 (0)	11 (2)	25 (5)	0 (0)	0 (0)	16 (14)	56 (10)	3 (3)
Baseline ECOG PS,* n* (%)									
0	155 (57)	210 (47)	216 (46)	144 (30)	85 (44)	24 (50)	41 (35)	283 (51)	34 (31)
1	99 (36)	214 (48)	231 (49)	265 (55)	94 (49)	19 (40)	70 (60)	246 (44)	66 (61)
2	20 (7)	19 (4)	27 (6)	77 (16)	13 (7)	5 (10)	5 (4)	25 (5)	8 (7)
Missing	7 (2)	2 (0)	1 (0)	1 (0)	0 (0)	0 (0)	0 (0)	0 (0)	0 (0)
Prior ASCT, *n* (%)	150 (53)	258 (58)	294 (62)	241 (49)	40 (21)	10 (21)	87 (75)	350 (63)	86 (80)
Number of prior LOT,* n* (%)									
1	33 (12)	205 (46)	221 (47)	1 (0)	52 (27)	0 (0)	0 (0)	289 (52)	0 (0)
2	208 (74)	139 (31)	142 (30)	25 (5)	66 (34)	10 (21)	32 (28)	156 (28)	2 (2)
3	40 (14)	100 (22)	64 (13)	167 (34)	30 (16)	10 (21)	34 (29)	76 (14)	21 (19)
≥ 4	0 (0)	1 (0)	48 (10)	294 (60)	44 (23)	28 (58)	50 (43)	33 (6)	85 (79)
IgG subtype MM,* n* (%)	144 (51)	255 (57)	253 (53)	261 (54)	81 (42)	29 (60)	55 (47)	306 (55)	89 (82)
Cytogenetic profile,^a^* n* (%)									
High	71 (25)	73 (16)	69 (15)	86 (18)	64 (33)	4 (8)	0 (0)	70 (13)	27 (25)
Standard	126 (45)	155 (35)	270 (57)	290 (60)	123 (64)	44 (92)	90 (78)	359 (65)	72 (67)
Missing	84 (30)	217 (49)	136 (29)	111 (23)	5 (2.6)	0 (0)	26 (22)	125 (23)	9 (8)
ISS stage, *n* (%)									
I	121 (43)	221 (50)	186 (39)	165 (34)	95 (49)	15 (31)	57 (50)	268 (48)	25 (23)
II	95 (34)	141 (32)	185 (39)	174 (36)	64 (33)	21 (44)	32 (28)	176 (32)	43 (40)
III	65 (23)	82 (18)	104 (22)	147 (30)	33 (17)	12 (25)	24 (21)	110 (20)	40 (37)
Missing	0 (0)	1 (0)	0 (0)	1 (0)	0 (0)	0 (0)	3 (3)	0 (0)	0 (0)

^a^Cytogenetic profile definition may vary across studies

*ASCT* autologous stem cell transplant, *ECOG PS* Eastern Cooperative Oncology Group performance status, *EMD* extramedullary disease, *IgG* immunoglobulin G, *IQR* interquartile range, *ISS* International Staging System, *LOT* line of therapy, *MM* multiple myeloma, *RRMM* relapsed/refractory multiple myeloma

Inclusion of three published studies gave a total of 228 patients with EMD (including both EMD and paramedullary disease) (Table S1) for the sensitivity meta-analysis; due to small patient numbers, data were pooled across the IKEMA and ICARIA studies. The baseline characteristics for patients in each of the three published studies are described in the original publications [32, 33].

## Meta-regression analysis in patients with versus without EMD

### ORR

**Fig. 1:**
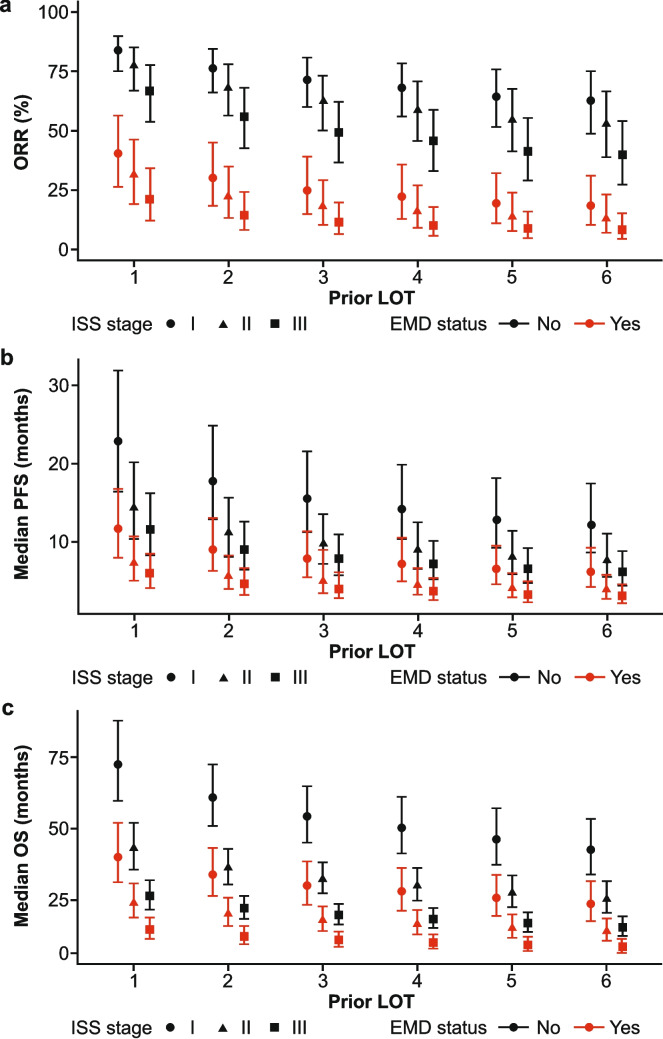
Estimates of pooled (**a**) ORR, (**b**) median PFS, and (**c**) median OS in patients with RRMM by EMD status, number of prior LOT, and ISS stage. Pooled ORR, pooled median PFS, and pooled median OS were estimated using a random-effects model and stratified by EMD status, number of prior LOT, and by ISS stage. Across all patients, age was held constant to the mean of 64 years. Data points are shown with 95% credible intervals. EMD, extramedullary disease; ISS, International Staging System; LOT, line of therapy; ORR, overall response rate; OS, overall survival; PFS, progression-free survival; RRMM, relapsed/refractory multiple myeloma

In the base model, a pooled ORR of 20.7% (95% CI, 11.7–33.9) was estimated in patients with EMD compared with a pooled ORR of 66.2% (95% CI, 53.0–77.4) in patients without EMD. The odds ratio was 0.13 (95% CI, 0.09–0.20), indicating that the likelihood of response was 87% lower in patients with EMD versus patients without EMD. Following adjustment for age, prior LOT, and ISS stage (Fig. 1a), the same results for pooled ORR were observed with an odds ratio of 0.13 (95% CI, 0.09–0.20). Pooled ORR was consistently higher in patients without EMD compared with patients with EMD who had similar ISS stage and number of prior LOT. Pooled ORR decreased in all patients who had higher ISS stages and decreased as the number of prior LOT received by a patient increased (Fig. 1a).

### PFS

In the base model, a pooled median PFS of 6.3 (95% CI, 4.2–9.5) months was estimated in patients with EMD compared with a pooled median PFS of 12.9 (95% CI, 8.8–18.8) months in patients without EMD. The hazard ratio was 1.95 (95% CI, 1.63–2.32), indicating that patients with EMD were approximately twice as likely to progress or die compared with patients without EMD. Following adjustment for age, prior LOT, and ISS stage (Fig. 1b), a hazard ratio of 1.92 (95% CI, 1.61–2.29) was observed for pooled median PFS, consistent with results of the base model analysis. Pooled median PFS was generally higher in patients without EMD compared with patients with EMD who had similar ISS stage and number of prior LOT, although overlapping 95% CIs were observed within each group. Pooled median PFS decreased in all patients who had higher ISS stages and decreased as the number of prior LOT received by a patient increased (Fig. 1b).

### OS

In the base model, a pooled median OS of 21.0 (95% CI, 15.9–27.9) months was estimated in patients with EMD compared with a pooled median OS of 39.0 (95% CI, 31.0–48.5) months in patients without EMD. The hazard ratio was 1.87 (95% CI, 1.53–2.26), indicating that patients with EMD were approximately 1.9 times more likely to die due to their disease compared with patients without EMD. Following adjustment for age, prior LOT, and ISS stage (Fig. 1c), a hazard ratio of 1.86 (95% CI, 1.51–2.27) was observed for pooled median OS, consistent with results of the base model analysis. Pooled median OS was consistently higher in patients without EMD compared with patients with EMD who had similar ISS stage and number of prior LOT. Pooled median OS decreased in all patients who had higher ISS stages and decreased as the number of prior LOT received by a patient increased (Fig. 1c).

### DOR

In total, 39 patients with EMD and 1824 patients without EMD achieved a partial response or better, respectively; these patients were included in the DOR analysis. In the base model, a pooled median DOR of 16.8 (95% CI, 10.3–27.4) months was estimated in patients with EMD compared with a pooled median DOR of 18.6 (95% CI, 13.3–25.6) months in patients without EMD. The hazard ratio was 1.12 (95% CI, 0.73–1.63). While this hazard ratio suggests that patients with EMD are approximately 1.1 times more likely to progress after achieving a response compared with patients without EMD, the observed difference is not significant, as the null value of 1 is contained within the 95% CI. Following adjustment for age, prior LOT, and ISS stage (Fig. S1), a hazard ratio of 1.18 (95% CI, 0.78–1.72) was observed for pooled median DOR, consistent with results of the base model analysis. Pooled median DOR was similar in patients with EMD and without EMD who had similar ISS stage and number of prior LOT. Pooled median DOR decreased in all patients who had higher ISS stages and decreased as the number of prior LOT received by a patient increased (Fig. S1).

### Meta-analysis in patients with EMD only

Results of the meta-analysis (with no adjustment for covariates) and sensitivity meta-analysis (including published studies) among patients with EMD were consistent with the meta-regression analysis, demonstrating robustness across all analyses, and showed poor outcomes in patients with EMD. Among the 228 patients with EMD who were included in the sensitivity meta-analysis, comprising both historical and published clinical studies, the pooled estimate for ORR was 24.8% (95% CI, 16.2–34.4) (Fig. S2a). The pooled estimate for median PFS was 6.0 (95% CI, 3.6–9.9) months (Fig. S2b) and for median OS was 20.4 (95% CI, 15.0–28.7) months (Fig. S2c).

## Discussion

In patients with RRMM who received standard treatments (PIs, IMiDs, and anti-CD38 mAbs), this meta-regression analysis showed patients with EMD were 87% less likely to respond to standard-of-care treatment regimens and twice as likely to progress or die compared with patients without EMD; results for these endpoints were consistent following adjustment for the baseline prognostic variables of patient age, number of prior LOT, and ISS stage. Results from the sensitivity meta-analysis also suggested that patients with EMD had poor outcomes on standard treatment regimens.

Within the historical and published clinical studies eligible for inclusion in this analysis, the numbers of patients with EMD were relatively small. A Bayesian random-effects model was therefore selected as the primary meta-analytic approach, allowing outcomes to be pooled across clinical studies and increasing the patient numbers available for analysis [30]. This approach provided higher precision in outcome estimates in patients due to use of aggregated estimates across studies while accounting for variability due to cross-study population differences. In addition, this model allowed for flexibility in specifying both binary (ORR) and time-to-event (PFS, OS, and DOR) outcome distribution models, as well as flexibility in calculating quantities of interest using posterior distributions [34].

Although patients with EMD had a lower pooled ORR than patients without EMD, there was no noticeable difference in pooled median DOR among these patient groups, as observed by overlapping 95% CIs, indicating that DOR in responders with EMD was comparable to DOR in responders without EMD. This finding is similar to other reported data in this patient population [14, 21] and highlights the importance of achieving a response as the most important factor impacting outcomes for patients with EMD. It should also be noted that as DOR analysis is limited only to patients who achieved a response (vs. an analysis in the intention-to-treat patient population), this may incur inherent bias, and results should be interpreted with caution.

The results of our analyses are generally consistent with other clinical study analyses in patients with EMD. In a retrospective, descriptive analysis, patients with versus without EMD had numerically lower ORRs (0.0%–50.0% vs. 42.7%–92.8%) and were more likely to progress and/or die during the first year of treatment [35]. Similarly, in a prospective analysis of patients with triple-class exposed RRMM, patients with EMD (both soft tissue plasmacytomas and paramedullary disease) versus patients without EMD had lower ORRs (24.1% vs. 33.3%), shorter median PFS (2.7 vs. 5.1 months), and shorter median OS (7.2 vs. 15.5 months); however, it should be noted that this patient population was more heavily pretreated than patients in the current meta-regression analysis [24]. While exact mechanistic drivers of EMD are not yet fully understood, several biological mechanisms may contribute to the consistently poorer outcomes observed in patients with versus without EMD [36, 37]. For example, when compared with bone marrow-restricted MM, EMD is often characterized by a reduction in adhesion molecules and an increase in extracellular matrix degrading enzymes, such as heparanase [3, 38], which may contribute to tumor spread and lower ORR in these patients. Furthermore, EMD lesions have been shown to exhibit a high level of genomic instability and heterogeneous expression of potential target antigens (e.g., GPRC5D and BCMA) [36, 37]. Taken together, these characteristics may contribute to therapeutic resistance and suboptimal outcomes in patients with versus without EMD.

There is currently no consensus definition for EMD, and many clinical studies do not clearly define EMD criteria, which complicates data interpretation [15]. For inclusion in the meta-regression analysis and meta-analysis, patients were required to have soft tissue plasmacytomas that were noncontiguous with bone; this approach is in line with a recent expert consensus review that defined “true” EMD as organ infiltration and soft tissue plasmacytomas that are noncontiguous with bone [14]. Patients with soft tissue plasmacytomas are known to have poorer outcomes than patients with paramedullary disease [16, 17, 39]. Thus, while results from this meta-regression analysis demonstrated outcomes in patients with “true” EMD to be poorer versus patients without EMD, these outcomes are also likely to be poorer than outcomes observed in patients with paramedullary disease who were not included in this meta-regression analysis.

While clinical studies with novel agents in RRMM, including bispecific antibody and CAR-T cell therapies, were not included in the current analysis and represent additional therapeutic options for patients with EMD [40–45], our findings are broadly in line with emerging real-world data for these novel agents, where patients with EMD have consistently poorer outcomes. Following treatment with novel agents, ORR may be higher than the ORR observed in our meta-regression analysis of patients who received standard treatments. Treatment with bispecific antibodies has shown ORRs of 31.7%–48.5% in patients with EMD (including both EMD and paramedullary disease) versus 68.3%–74.1% in patients without EMD [41, 42, 45]. Treatment with CAR-T cell therapies has shown ORRs of 52%–58% in patients with EMD versus 82%–96% in patients without EMD [43, 44]. While CAR-T cell therapies have limitations, such as manufacturing complexity that limits patient eligibility, off-the-shelf bispecific antibodies may be more widely available and accessible for many patients [46, 47]. In a study of talquetamab, a bispecific antibody that targets G protein–coupled receptor family C group 5 member D on myeloma cells, in combination with teclistamab, a bispecific antibody that targets B-cell maturation antigen on myeloma cells, patients with EMD (vs. patients without EMD) had a promising ORR of 61% (vs. 80%), an 18-month DOR rate of 82% (vs. 86%), and a 12-month PFS rate of 53% (vs. 74%) [40]. In our meta-regression analysis, the median PFS of patients with EMD was 6.3 months (versus 12.9 months in patients without EMD), which is consistent with median PFS observed following treatment with approved T-cell redirecting therapies, including CAR-T therapies and bispecific antibodies (< 6 months) [36]. Together, these findings indicate novel therapies may be beneficial in this patient population; however, longer follow-up is required to fully delineate potential long-term efficacy outcomes.

There are some limitations to these analyses. Not all FDA-approved therapies for RRMM were included, only studies that reported outcomes stratified by EMD status were included, and some studies did not report data for all outcomes (e.g., the STORM study did not report PFS or OS). Individual patient data were not available in the published studies and therefore were not included in the meta-regression analysis. Additional variables, such as cytogenetic risk or Eastern Cooperative Oncology Group performance status, were not included in the meta-regression modeling and analysis due to the proportion of missing data for cytogenetic risk and that most patients, across studies, had an Eastern Cooperative Oncology Group performance status of 0 or 1, indicating high functional ability. Patients with EMD could also have had other high-risk factors, such as high-risk cytogenetics, which may have contributed to response outcomes observed in this study. Exposure status was not specified per study inclusion criteria and therefore varied from 1 to ≥ 4 prior LOT; this may preclude direct comparisons to other patient populations (i.e., those defined as triple-class exposed only). However, these results do provide a benchmark to which more heavily treated patient populations may be compared, alongside the potential generalization that outcomes may be even worse in entirely heavily treated patient populations.

In conclusion, these results utilizing approved standard treatment regimens in patients with RRMM confirmed suboptimal outcomes in patients with EMD compared with patients without EMD, emphasizing the continued unmet clinical need in this patient population. Novel therapies, including CAR-T therapies, bispecific antibodies, and dual-antigen targeting approaches, are currently being investigated for the treatment of patients with RRMM and EMD; these approaches may circumvent antigen escape and improve patient outcomes.

## Supplementary Information

Below is the link to the electronic supplementary material.

Supplementary file1 (DOCX 321 KB)

## Data Availability

The data sharing policy of Johnson & Johnson is available at [https://www.jnj.com/innovativemedicine/node/87]. As noted on this site, requests for access to the study data can be submitted through Yale Open Data Access (YODA) Project site at [https://yoda.yale.edu/].
