# Supplementary material for: Outcomes in patients with relapsed/refractory multiple myeloma with extramedullary disease: a meta-analysis
Source: Ann Hematol. 2025 Dec 3;104(12):6263–74. doi: 10.1007/s00277-025-06705-3 (PMC12764547; doi:10.1007/s00277-025-06705-3)
Supplement: Supplementary file 1 — Supplementary file1 (DOCX 321 KB) [file 277_2025_6705_MOESM1_ESM.docx]

# Supplementary Materials

# Supplemental Tables

## **Table S1** Treatment regimens across historical and published clinical studies

| Treatment | Meta-regression analysis (EMD and  non-EMD) in historical studies only | Meta-analysis  (EMD only) in historical studies only | Sensitivity  meta-analysis including published studies  (EMD^a^ only) |
| --- | --- | --- | --- |
| Daratumumab monotherapy (SC) | 263 | 17 | 17 |
| Daratumumab monotherapy (IV) | 553 | 40 | 40 |
| Daratumumab, carfilzomib, dexamethasone (DKd) | 312 | 13 | 13 |
| Daratumumab, lenalidomide, dexamethasone (DRd) | 286 | 9 | 9 |
| Daratumumab, pomalidomide, dexamethasone (DPd) | 151 | 15 | 15 |
| Daratumumab, bortezomib, dexamethasone (DVd) | 392 | 24 | 24 |
| Carfilzomib, dexamethasone (Kd)^b^ | 154 | 8 | 15 |
| Pomalidomide, dexamethasone (Pd)^b^ | 153 | 8 | 18 |
| Lenalidomide, dexamethasone (Rd) | 283 | 6 | 6 |
| Bortezomib, dexamethasone (Vd) | 317 | 18 | 18 |
| Isatuximab, carfilzomib, dexamethasone (Isa-Kd)^b^ | NA | NA | 12 |
| Isatuximab, pomalidomide, dexamethasone (Isa-Pd)^b^ | NA | NA | 14 |
| Selinexor, dexamethasone | NA | NA | 27 |
| Total patient number in analyses | **2864** | **158** | **228** |

Abbreviations of treatment regimens are included in parenthesis where appropriate

^a^Definitions varied across published studies and included both EMD and paramedullary disease

^b^Due to small patient numbers, results were pooled for Isa-Kd and Isa-Pd and included as one treatment in the meta-analysis. Results from the Pd and Kd control arm treatments were similarly pooled and included as one treatment in the meta-analysis

EMD, extramedullary disease; IV, intravenous; NA, not applicable as not included in the primary analysis; SC, subcutaneous

# Supplemental Figures

## **Fig. S1** Estimates of pooled median DOR in patients with RRMM by extramedullary disease EMD status, number of prior LOT, and ISS stage


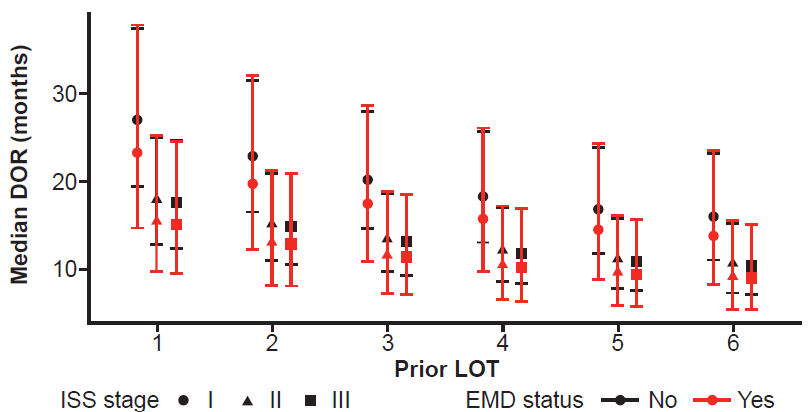
Pooled median DOR was estimated using a random-effects model and stratified by EMD status, number of prior LOT, and by ISS stage. Across all patients, age was held constant to the mean of 64 years. Data points are shown with 95% credible intervals
DOR, duration of response; EMD, extramedullary disease; ISS, International Staging System; LOT, line of therapy; RRMM, relapsed/refractory multiple myeloma

## **Fig. S2** Pooled estimates of (a) ORR and median (b) PFS and (c) OS in patients with RRMM with EMD

**
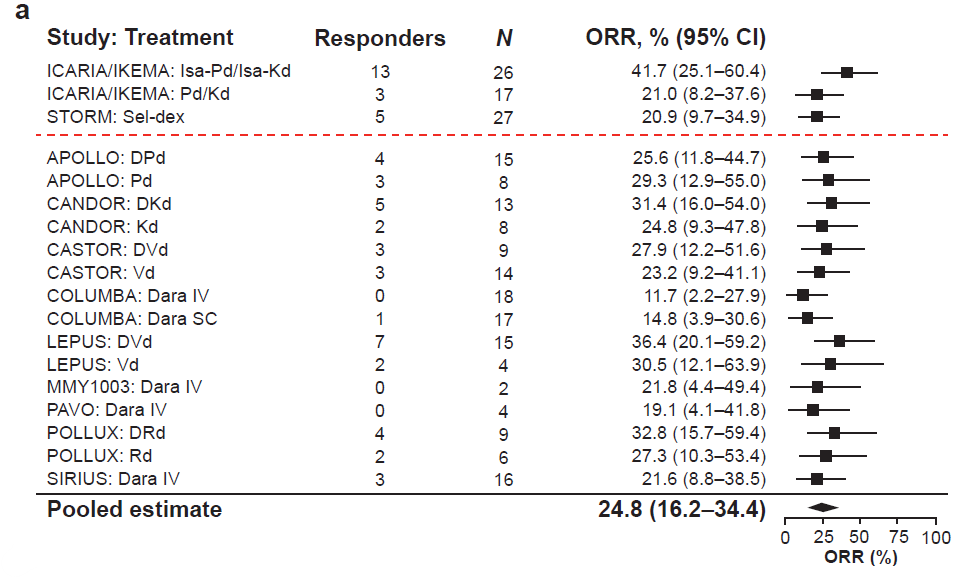
**


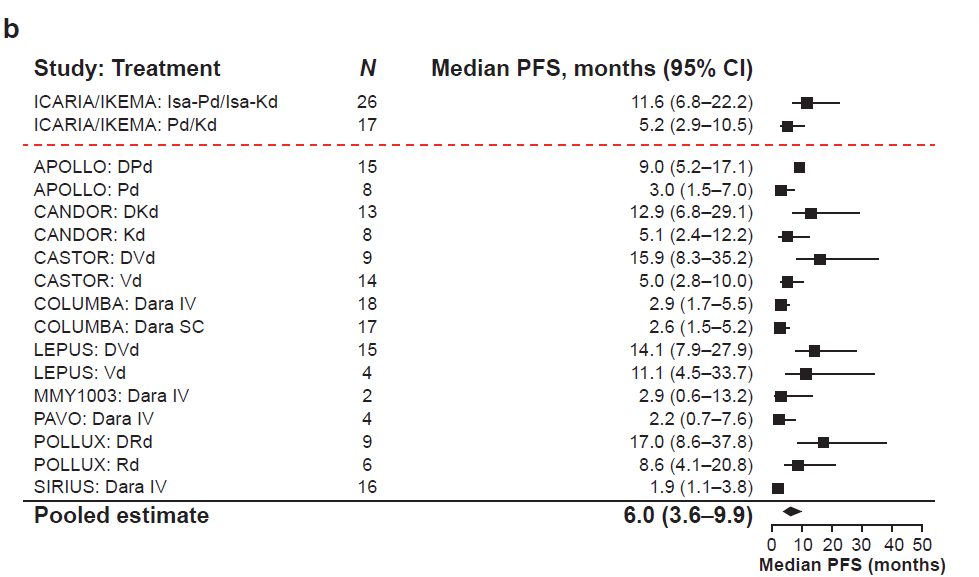


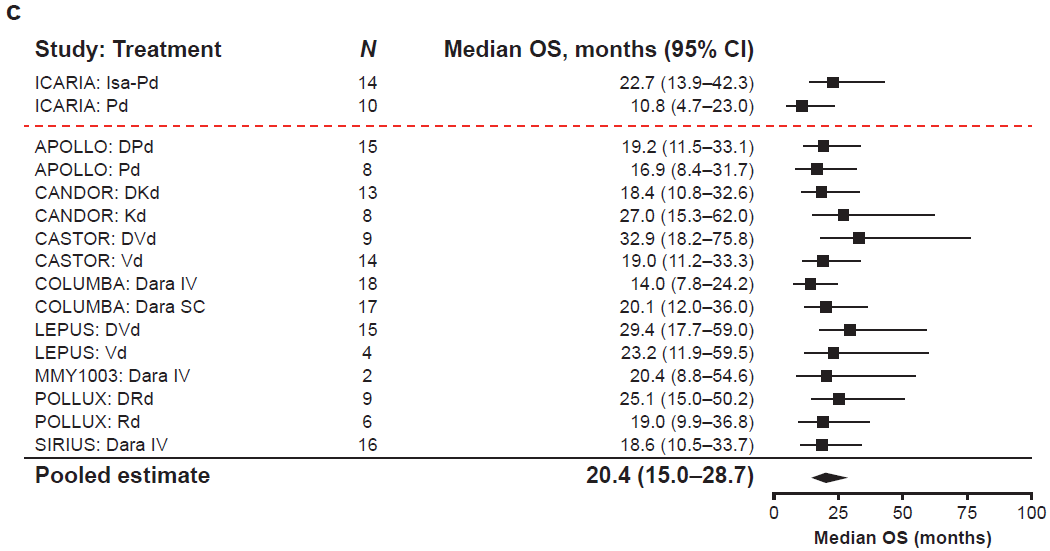


Historical studies included EMD defined as plasmacytomas noncontinguous with bone (“true” EMD). Definitions of EMD varied across published studies and included both EMD and paramedullary disease

Pooled estimates for ORR, PFS, and OS are estimated using the random-effects model. In the top panel, responders (patients who achieved better than partial response [≥PR]) and *N* are observed study and treatment counts. In the middle and bottom panels, *N* are observed study and treatment counts. The STORM study did not include progression-free survival in the published literature and therefore was not included in this analysis. The IKEMA and STORM studies did not report overall survival in the published literature and therefore were not included in the analyses

DKd, daratumumab, carfilzomib, dexamethasone; DPd, daratumumab, pomalidomide, dexamethasone; DRd, daratumumab, lenalidomide, dexamethasone; DVd, daratumumab, bortezomib, dexamethasone; EMD, extramedullary disease; IKd, isatuximab, carfilzomib, dexamethasone; IPd, isatuximab, pomalidomide, dexamethasone; IV, intravenous; Kd, carfilzomib, dexamethasone; ORR, overall response rate; OS, overall survival; Pd, pomalidomide, dexamethasone; ORR, overall response rate; OS, overall survival; PFS, progression-free survival; PR, partial response; Rd, lenalidomide, dexamethasone; RRMM, relapsed/refractory multiple myeloma; SC, subcutaneous; sel-dex, selinexor, dexamethasone; Vd, bortezomib, dexamethasone

# Supplementary Information

## **Methods**

## **Analyses**

The default shape parameter for the Weibull distribution was used [Gamma(0.01, 0.01)]. For analysis of outcomes of interest, weakly informative priors [Student’s T(df = 3, mu = 0, sigma = 2.5)] were used. Weakly informative priors were selected for use in this analysis to ensure a crucial balance in maintaining the stability of the statistical model without constraining the results. As such, weakly informative priors allowed the statistical model to remain flexible enough for the data to shape the posterior distribution, while also providing a layer of robustness against model instability and unrealistic estimates, especially when data are sparse or limited. Models were fit using the Stan-based “brms” R package. Model fit was evaluated by examination of the convergence and mixture of chains, which were confirmed by R-hat metrics and visual inspection, respectively (data not shown). Posterior predictive checks informed model reparameterization (data not shown).
